# Study on the Public Perception of “Community-Owned Dogs” in the Abruzzo Region, Central Italy

**DOI:** 10.3390/ani10071227

**Published:** 2020-07-19

**Authors:** Alessandra Paolini, Sara Romagnoli, Maria Nardoia, Annamaria Conte, Romolo Salini, Michele Podaliri Vulpiani, Paolo Dalla Villa

**Affiliations:** Istituto Zooprofilattico Sperimentale dell’Abruzzo e del Molise “G. Caporale”, via Campo Boario, 64100 Teramo, Italy; a.paolini@izs.it (A.P.); romagnolisara.vet@gmail.com (S.R.); maria.nardoia@gmail.com (M.N.); a.conte@izs.it (A.C.); r.salini@izs.it (R.S.); p.dallavilla@izs.it (P.D.V.)

**Keywords:** community-owned dogs, public perception, stray dogs, free-roaming dogs

## Abstract

**Simple Summary:**

The present study was conducted in the Abruzzo Region, central Italy, to investigate the public perception towards “community-owned dogs” (CODs); i.e., whether their presence is perceived as a problem or a benefit by the local communities. The data were collected by both direct interviews and an online survey, based on a questionnaire developed by a multidisciplinary team. The questionnaire was distributed in 31 municipalities sampled on the basis of the urbanization rate, and 497 people were interviewed over 9 months. The majority (83%) believed that a greater commitment is needed to involve the local communities on issues regarding CODs. The findings of this study highlighted the general difficulty for people to distinguish stray dogs from CODs that are not fully known, as evidenced by the fact that 59% of the respondents are not aware of the Regional Law that defines and regulates the presence of the CODs.

**Abstract:**

The Abruzzo Regional Law Nr. 47/2013, following a circular from the Italian Ministry of Health and OIE recommendations, allows the local municipalities to release free-roaming dogs (FRDs) caught on the territory once the local veterinary services (LVSs) have rated the dogs as unowned and not aggressive, have neutered them, as well as identified them through a microchip and a visible collar. The responsibility of these “community-owned dogs” (CODs) falls under the mayor of the local municipality that can entrust their custody to qualified people. The present study was conducted in the Abruzzo region, located in central Italy, to investigate public perception towards CODs, and in particular whether their presence is perceived as a problem or a benefit by the local communities. The data were collected by both direct interviews and an online survey, based on a questionnaire developed by a multidisciplinary team. The questionnaire was distributed in 31 municipalities sampled on the basis of the urbanization rate, and 497 people were interviewed over a 9-month period. More than half of the respondents (54%) stated that CODs can contribute to the control of stray dogs. The majority (83%) believed that a greater commitment is needed to involve the local communities on issues regarding CODs. The findings of this study highlighted the general difficulty for people to distinguish stray dogs from CODs that are not fully known, as evidenced by the fact that 59% of the respondents did not know the aforementioned Regional Law that defines and regulates the presence of the CODs.

## 1. Introduction

Under the Italian Framework Law Nr. 281/1991 [1], on the protection of companion animals and the prevention of stray animals, in Italy it is not legal to euthanize dogs and cats unless they are proven to be dangerous or incurably ill. In Circular No. 5, dated 14 May 2001 [2], the Italian Ministry of Health states that excessive canine proliferation, determined by the uncontrolled reproduction of free-roaming dogs (FRDs), has significantly increased the presence of stray dogs and has pushed local administrators to seek alternative solutions to kennels, such as the “neighbourhood dog” or “community-owned dog” (COD).

The purpose stated in the circular is that dogs can survive given their remarkable ability to adaptation and considering that the neighbourhood inhabitants, without the burden of the dog ownership responsibility, will endeavour to procure them food and shelter [2].

The “community-owned dog” (COD) is defined as an animal recognized as unowned; regarded non-hazardous by the public veterinary service; sterilized and identified by a microchip and fitted with a collar that allows identification at a distance; registered in the regional register of dogs and released in the place where it was captured; and entrusted to qualified personnel identified by the territorially competent municipality, to protect the health and welfare and recognizing the right to be a free animal.

In the Abruzzo region, the management of CODs is regulated by Regional Law No. 47/2013 [3]. In Europe, there is a limited amount of national regulations for the protection of community dogs. To date, only 24 European countries are party to the Council of Europe Convention for the Protection of Pet Animals (CoE, 1987) [4], aiming at promoting the welfare of companion animals and ensure minimum standards for their treatment and protection. As a convention, it has been ratified by Italy in 2011 and it represents a voluntary commitment to implement these rules at the national level. In 2009, the World Organization for Animal Health also adopted animal welfare standards applicable to stray dog population control as part of the Terrestrial Animal Health Code. The OIE standards are not immediately binding but still represent a fundamental tool to fight against zoonotic diseases and other nuisances produced by FRDs. According to these recommendations, once stray dogs are released to the territory as CODs, they should be returned to a place that is as near as possible to the place of capture, their welfare should be regularly monitored and they should be easily identifiable on sight (e.g., visible collar) to avoid unnecessary recapture [5]. It also recommends that, if this method is adopted, raising awareness of the program within the local community should be addressed to ensure understanding and support. To date, this matter is not governed by EU rules and the European Convention for the Protection of Pet Animals is not part of the EU legislation. How EU member states implement national legislation on these matters remain under their sole competence.

The present study aims to investigate, five years after the application of Abruzzo Regional Law No. 47, the awareness of the Abruzzo residents of the CODs and if the CODs are tolerated by local communities or if inhabitants complain of any disturbance they may cause (e.g., noise, faecal pollution and bite injuries), and to survey the opinion of the caretakers.

## 2. Materials and Methods

The present study was carried out from June 2018 to February 2019 through an anonymous questionnaire to assess the perceptions of Abruzzo residents toward CODs.

### 2.1. Sample Size Calculations

In order to calculate the minimum sample size, a probability of respondents of 50%, a confidence level of 95% and a sampling error of 5% were considered:

(1) n=p·(1−p)·Zα22e2=0.5·(1−0.5)·1.9620.052=384

At the moment of the survey, the available data reported a total population of the Abruzzo region of 1,322,247 inhabitants (ISTAT 2017).

The questionnaires were equally distributed in 10% of the 305 municipalities of the Abruzzo region, divided into 3 urbanization categories: 128 interviews in 15 municipalities with a low urbanization rate; 128 interviews in 15 municipalities with a medium urbanization rate; and 128 interviews in Pescara, the only high urbanization rate municipality present in the Abruzzo region.

### 2.2. Participants and Recruitment

Participation in this project was entirely voluntary, and consent was understood as implicitly given.

The questionnaire was anonymous and no personal information or that allowed to identify the participants in any way was collected. Moreover, there were no foreseeable risks in taking part in the study. For this reason, the processing of the information collected does not fall within the application of the legislation on the protection of personal data (Regulation EU 2016/679 (GDPR)). Only people over 18 years of age were considered eligible to complete the survey.

Participants in the questionnaire were provided with detailed information sheets that were written in an understandable language and terms, including the objectives, methods and implications of the research, and the nature of their participation. Considering that the questionnaire was anonymous and that the results were shown in an aggregated form, the results and potential misuse of the research/results methodology or ethics of this study do not harm any religious, ethnic, or racial group.

Two methods were adopted to collect data; questionnaires were administered through an in-person interview or an online version.

For the second method, participants were recruited using a “virtual snowballing” technique [6], which involved requesting personal and professional contacts of the research team by email or social media (e.g., Facebook.com or WhatsApp) to complete the survey and forward this request to their personal and professional contacts. This method is widely used in the literature and was chosen in order to enrol an adequate number of respondents that were interested in the topic of the study. The demographic characteristics of all the respondents were systematically collected. Those who volunteered to participate clicked on a link in the message that linked directly to the survey site (https://docs.google.com/forms/u/0/).

### 2.3. Questionnaire Design

The survey was constructed evaluating prior literature on the topic [7,8,9,10,11].

The questionnaire was developed through a multidisciplinary approach, with the collaboration of veterinarians, an agronomist, animal behaviour experts, a psychologist, and statisticians.

An undeclared pre-test was submitted to 100 persons. It was revised (question sequence, word choices, etc.) according to the inputs from the above experts; the people involved in developing the survey were the same who pre-tested it.

The final questionnaire included an introduction explaining the purpose of the study, how the information gathered would be used, assurance that the survey would be anonymous, and the contact details of the primary researcher.

The semi-structured questionnaire consisted of twenty-eight questions (open or multiple-choice questions) divided into three sections. Section 1 aimed to collect demographic data about the participants and consisted of thirteen multiple choice questions regarding their gender, age, education, and area of residence (town/village, outskirts or countryside). Interviewees were also asked if they owned a pet and which one. Ranges were provided for age and education.

The second part of the survey focused on CODs; at the beginning of this section, an explanation of the expression “community-owned dog” was given. The participants were asked if they knew CODs, their feelings towards them, and if they took care of these animals.

In the third section, the participants were asked if they considered CODs to be a problem for public health, personal safety, environmental hygiene, other animal safety, or an effective measure to control the stray dog population. It was also asked what might be done about free-roaming dogs (with specific answer choices listed).

The 28 questions that made up the questionnaire are listed in Table S1.

### 2.4. Data Analysis

To investigate possible associations between the data of the respondents (residence area, possession of pets) and the respondents’ answers, a chi-square test was applied. When the expected frequencies were less than 5, a Fisher exact test was used [12]. In both cases, the test is considered statistically significant when the *p*-value is <0.05. For the chi-square test calculation, XLSTAT Version 2013.2.04 AddsInSOFT ^TM^ was used.

## 3. Results

The percentage of the responses to each question and the results of the data analysis are provided in Table S1.

**Figure 1:**
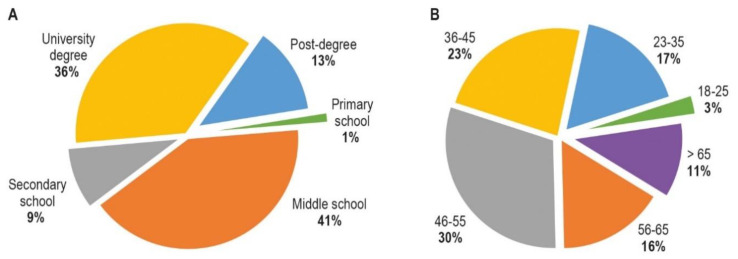
The demographic characteristic of all respondents (*n* = 497) education level: (**A**) and age (**B**).

There were 497 respondents (300 females and 197 males). The demographic characteristics (age and education level), collected in Section 1 of the questionnaire (questions 1 to 8), are provided in Figure 1.

Section 2 of the questionnaire referred to more specific issues on CODs and the human–animal relationship. In particular, at Question 9, “Do you know about the existence of CODs in the Abruzzo region and that this figure is regulated by a regional law?”, 59% of respondents answered “No”.

About half of all the questionnaire participants (250/497, 50,3%) were in favour of CODs as a control measure for stray dogs; considering that 31 people did not answer this question, about 54% (250/466) of those who responded to this specific question answered to support this solution, 133 respondents were against it, 45 were indifferent, and 38 indicated “other” without adding other observations.

**Figure 2:**
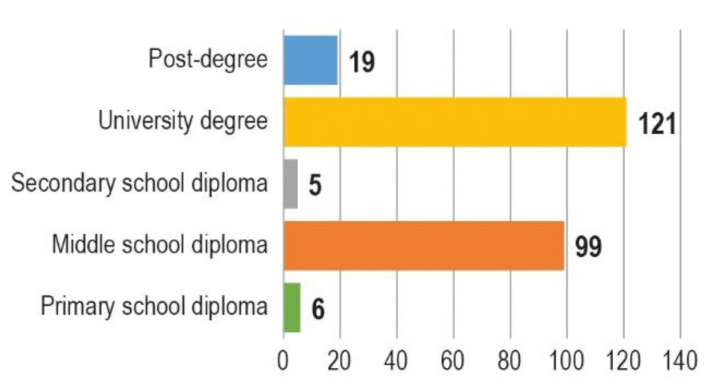
Educational level of the respondents who consider community-owned dogs (CODs) an effective measure to control stray dog populations (*n* = 250).

**Figure 3:**
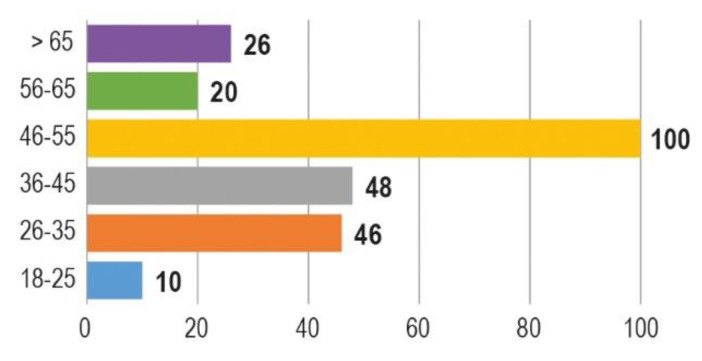
Age of respondents who consider CODs an effective measure to control stray dog populations (*n* = 250).

As concerning the 250 respondents in favour of CODs, they were 171 females (68%) and 79 males (32%), the majority with a university degree (121/250, 48,4%) or a middle school diploma (99/250,39,6%) (Figure 2). Most part of the respondents in favour of CODs belonged to the age group 46–55 years old (100/250, 40%) (Figure 3).

**Figure 4:**
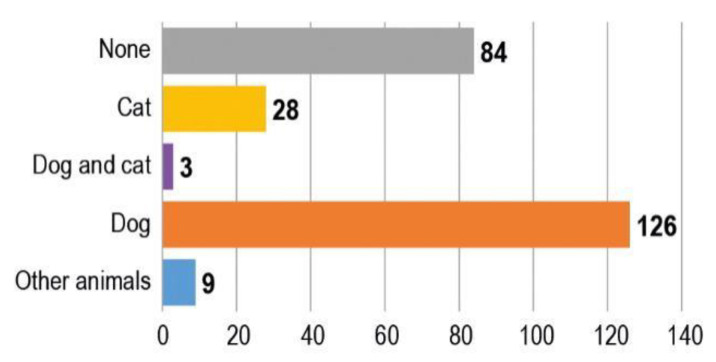
Pet owned by respondents who consider CODs an effective measure to control stray dog populations (*n* = 250).

Figure 4 shows how many of the respondents in favour of CODs owned a pet and which animal they had.

Those who are unfavourable to CODs indicated as alternative solutions adoption and responsible ownership promotion (48%), birth control (41%), as well as building new kennels (6%).

Twenty-eight respondents, all-female, declared to take care of CODs, 57% monthly and the rest weekly; 75% of them (21/28) were dog owners; and 10 of them belonged to the class age 36–45 years old, 8 to the class 26–35, 4 to the class 46–55, and the rest was over 65 years old. The main education level was the high school (13/28), followed by a degree or post-graduate degree (9/28) and finally elementary school (6/28).

Table S2 shows the number of respondents who perceived CODs as a problem, relating their opinion to the area in which they lived. A statistically significant association was found only for Question 24, “In your opinion, could CODs be a problem for personal safety?”: there were 326 respondents out of 425 who believed that CODs were not a personal safety problem; the association among respondents residence area and answers was significant (chi-square = 16.67, *p*-value < 0.01), highlighting differences in the frequencies (see Supplementary Materials).

Table S3 in the Supplementary Materials shows the number of respondents who perceived CODs as a problem or not, relating their opinion to the possession of a pet. A statistically significant association was found for three of the four perceived risks: public health (Fisher exact test, *p*-value < 0.01), environmental hygiene (Fisher exact test, *p*-value < 0,05), and other animals’ health and safety (Fisher exact test, *p*-value < 0,05). Respondents who owned a pet were more likely to believe that CODs were not a risk. Of the 349 respondents who believed that CODs were not a problem for public health, 271 owned a pet.

A high percentage of the respondents (410/497, 83%) thought that people should be better educated and involved in COD issues; 59% of respondents reported that they were unaware of the existence of CODs in the Abruzzo region and that this figure was regulated by the regional law.

## 4. Discussion

The present study aims to investigate the public perception of CODs in the Abruzzo region five years after the application of the Abruzzo Regional Law No. 47/2013 [3], which regulates their management. The presence of CODs in the territory is only one of the possible measures put in place in the different countries to counter/reduce the presence of FRDs, i.e., dogs free to roam, mate, and reproduce [13,14]. In fact, it is estimated that there are about 700 million animals in the FRD population and, considering this number, that represents about 75% of the dogs in the world [6]; it is, therefore, easy to understand the extent of the phenomenon and the different approaches used to manage it in the best way.

In fact, to have FRDs in one’s territory generates a series of problems, which have significant implications on public health (e.g., the transmission of rabies and other zoonotic pathogens) and animal welfare [13]. The solutions adopted for the management of this problem must take into account both economic, health, ethical, religious, and legal aspects [15]. Currently, the FRD problem is a worldwide problem; there are three main approaches to manage this issue, namely, culling, sheltering, and fertility control, or a combination of these.

It should be pointed out that a constant change in public perception and sensitivity towards pets and a growing interest in their welfare [7] have led the most important international institutions to give indications on how to manage the phenomenon. In fact, dog population management, as a multifactorial issue, is part of the multidisciplinary concept of “One Health”, which requires an integrated approach that incorporates the animal, human, and environmental components, and promotes inter-professional collaboration [16]. International organisations, such as the World Health Organization, FAO, or OIE, have commented on this issue and some have also developed guidelines discouraging the use of culling and recommending alternative methods (e.g., registration and identification, vaccination, public education, and sterilization) [17,18].

In some countries, sheltering free-roaming dogs is the most common method of dog population control. On the other hand, fertility control can be achieved through surgical or chemical sterilization or contraception [14]. In particular, surgical sterilization through catch–neuter–release (CNR) of free-roaming dogs is the predominant method of fertility control. This method involves collecting free-roaming dogs and carrying out spay or castration surgery. [14]

In the European Union, there are also different approaches from country to country and in some cases, within their own national territory, different measures are applied to deal with the problem of the FRDs. While, on the one hand, the member states of the Council of Europe have recognized that, since 1987, man has a moral obligation to respect all living creatures and recognize the special links between man and pets, and that the link between animals and society is evolving and becoming more inclusive [19], on the other hand, unfortunately, there is no specific reference to the welfare of owned dogs or the welfare of FRDs or CODs. Indeed, as far as pets are concerned, EU legislation deals exclusively with the transport conditions and veterinary checks of pets for commercial purposes and trade in exotic species. All aspects of pet welfare are therefore dealt with in national legislation and differ from country to country [20]. To date, many European countries (Germany, Switzerland, Austria, Czech Republic, France, Italy, France, and United Kingdom) have adopted specific legislation or a code of conduct for the protection of pets [19]. However, so far there is no single mandatory legislation on the control of stray dogs in Europe and this leads to different approaches in the European Union [19].

Many European countries implement a trapping policy with euthanasia protocols for animals trapped after a 3- to 60-day detention period [20]. According to the Carodog website, “nine European Member States (plus the Spanish region of Catalonia) have already strictly prohibited the killing of healthy dogs. These countries are Austria, Bulgaria, Czech Republic, Germany, Greece, Italy, Lithuania, the Netherlands and Sweden” [21]. Since 2016, practicing euthanasia of healthy animals also has been forbidden in Portugal [22].

In some European countries (Bosnia-Herzegovina, Bulgaria, Greece, Malta, Serbia, Spain, and Italy), one approach is the above described “catch, neuter, release” of dogs (CNR) [17]. In these countries, the strategy of CNR is modelled on the “trap, sterilization and return” (TNR) of cats, and the Federation of Veterinarians of Europe considers this measure in its position paper on the management of stray dogs [23].

In Italy, there are about 600,000 stray dogs (Ministry of Health, 2012) [24], even if since 1991 the aforementioned law No. 281/1991 [1] aimed to prevent stray dogs, to punish the abandonment of the animals, and to entrust the local veterinary service (LVS) to sterilize stray dogs and cats. With the opportunity introduced by Circular No. 5, dated 14 May 2001 [2], six Italian regions, among which Abruzzo is one, adopted CODs as an alternative measure to control stray dog populations; however, as far as we know, there are no published data on the effectiveness of this measure nor surveys on the opinion of residents in these regions.

The economic and socio-cultural differences between the Italian regions explain the adoption of CODs as an alternative measure to stray dogs only in some of them, in addition to those indicated in the Framework Law No. 281. A COD is a means to reduce the number of stray dogs indicated as advantageous, especially considering the cost–benefit ratio [25]. Catch–neuter–vaccinate–release is a program that has been used successfully in some regions but may not work for other areas. The local situation should always be taken into consideration [23], as well as its reflection on the welfare of these animals. Each dog population management option should take into account different aspects, including ethical, socio-economic, political, and religious specificities of the local context, which will influence their acceptance [16].

We know that the COD option in a broader stray dog management program has advantages but also disadvantages. For example, in developing countries, most dogs are community dogs, affiliated to neighbourhoods rather than individual owners [18]. These animals are included by Slater [13] among strays, considering them almost owned. Most of the problems related to these dogs are therefore the same as for strays, with the main difference of potential increased access to sterilization programs and feed supply. In a previous study [7], which consisted of a telephone survey involving 396 residents in the province of Teramo (Abruzzo), 90% of the respondents believed that dogs and cats in the wild were a problem. The most commonly perceived problem was personal safety, followed by animal welfare, public health, and environmental hygiene [7].

If it is correct that the neighbourhood dog has the opportunity to live free and, for this reason, to show all normal behaviour, this dog has a higher chance of dying from an accident or poisoning than being abused. Considering the necessity of adequate feeding and veterinary cares, the welfare of CODs is strictly connected to the person or persons in charge. Therefore, it is essential that in addition to being required by law there is a profound acceptance of this animal in the community in which it lives.

From the analysis of the data collected in our study, it is clear that most of the interviewees say that they are in favour of using this measure as a tool for the control of stray dogs. The COD is usually not perceived as a possible problem for personal safety, environmental hygiene, or public health, particularly among city residents rather than agricultural areas residents. Interestingly, most of the respondents that appreciate CODs as a stray dog management measure own a pet. This result can be read from two points of view: one is that CODs are somehow perceived by the owners of animals as respectful of the animal itself (the dog is adopted and lives free rather than forced to live inside a kennel), and the other is that, by owning a pet, they would not perceive the presence of a dog well inserted in an urban context as a problem. It also seems important to stress how efforts to increase citizenship information about the neighbourhood dog emerge. Indeed, drafting a neighbourhood dog law without taking citizens’ perception of the problem into account could lead to mismanagement of the problem.

In the decade 2009–2018, as many as 7626 dogs were entered into the Abruzzo dog registry office as CODs released in the territory, and based on the official database, they are still alive and not adopted. Barnard et al. 2015 [26] report that no reliable estimates of the population of FRD in the province of Pescara (Abruzzo) are available. The results of our study show that a high percentage of respondents (59%) are not aware of the existence of the rules governing the management of CODs. A general difficulty of people to distinguish CODs from FRDs is assumed. According to the regional law, CODs should be remotely identifiable. However, in practice, with rare exceptions, no means are used to facilitate this differentiation, as reported by the LVSs (personal communication).

The absence of visible identification means that FRDs are sometimes mistaken for CODs, but FRDs have no guarantee of health status and are free to reproduce, increasing the number of strays. On the contrary, CODs can be confused with FRDs and can, therefore, be perceived as a more significant problem by citizens. Although OIE guidelines suggest the need for visible identification (e.g., collar), also to avoid unnecessary recapture [4], a study carried out in the province of Pescara (Abruzzo) confirms that a rather constant percentage of CODs is recaptured every year and this leads to unnecessary stress for the animals and avoidable expenditure of public resources [26]. For this reason, each COD should be constantly provided with a visible marking (collar, ear tag) to make it easily recognizable and monitored, even from a distance.

## 5. Conclusions

A COD is a measure to reduce stray dogs advantageously, mainly from the cost–benefit point of view [24]. According to our results, the management of this virtuous solution would need some improvements in the Abruzzo region.

In our study, we assume a general difficulty of residents in distinguishing CODs from FRDs. A total of 59% of the respondents were unaware of the existence of the rules governing the management of CODs but, after being informed, about 54% (250/466) answered to support this control measure.

A high percentage of the respondents (410/497, 83%) thought that people should be better educated and involved in COD issues.

In particular, the following efforts should be addressed: to inform Abruzzo residents about CODs as a low-cost measure of stray dog control, to enhance their awareness and support.

It would be advisable to reproduce this work in a few years to be able to evaluate whether, after implementing the suggested improvement actions, the public perception of CODs has changed and to which degree.

## Supplementary Materials

The following are available online at https://www.mdpi.com/2076-2615/10/7/1227/s1, Table S1: Questionnaire and responses percentage, Table S2: Association between the respondents who consider CODs a problem for the personal safety (Question 24), the Public health (Question 24.1), the environmental hygiene (Question n.24.2), the other animals’ health and safety (Question n.24.3) and their residence area, Table S3: Association between the respondents who consider CODs a problem for the personal safety (Question 24), the public health (Question 24.1), the environmental hygiene (Question n.24.2), the other animals’ health and safety (Question n.24.3) and their pet ownership.
