# Supplementary material for: Study on the Public Perception of “Community-Owned Dogs” in the Abruzzo Region, Central Italy
Source: Animals (Basel). 2020 Jul 19;10(7):1227. doi: 10.3390/ani10071227 (PMC7401527; doi:10.3390/ani10071227)
Supplement: Supplementary file 1 [file animals-10-01227-s001.zip › Supplementary Materials.docx]

Article

Supplementary Materials: Study on the Public Perception of “Community Owned Dogs” in the Abruzzo Region, Central Italy

**Alessandra Paolini, Sara Romagnoli, Maria Nardoia, Annamaria Conte, Romolo Salini, Michele Podaliri Vulpiani * and Paolo Dalla Villa**

**Table S1.** Questionnaire and responses percentage.

| ***Section 1*** | **GENERAL INFORMATION** | | |
| --- | --- | --- | --- |
| ***1. Gender*** | **M** | | 40% |
|  | F | | 60% |
| ***2. Age*** | 18–25 | | 3% |
|  | 26–35 | | 17% |
|  | 36–45 | | 23% |
|  | 46–55 | | 30% |
|  | 56–65 | | 16% |
|  | >65 | | 11% |
| ***3. Education level*** | Primary school | | 1% |
|  | Middle school | | 9% |
|  | Secondary school diploma | | 41% |
|  | University degree | | 36% |
|  | Post-degree (PhD, Master degree, etc.) | | 13% |
|  | Other __________________ | |  |
| ***4. Region of residence*** | **Abruzzo** | |  |
| ***5. Residential area*** | Town/Village | | 70% |
|  | Outskirts | | 19% |
|  | Country side | | 10% |
| ***6. Do you own pet?***  Multiple choice allowed | Yes, dog | | 59% |
|  | Yes, cat | | 9% |
|  | Yes, other animals _______________ | | 6% (other animals)  1% dog and cat |
|  | None | | 26% |
| ***7. If you own a dog, where does it come from?***  Multiple choice allowed | Adopted (from kennel/after a requisition) | | 21% |
|  | Rescued from the street | | 30% |
|  | Given by friends/relatives | | 37% |
|  | Purchased (in a pet-shop/on-line) | | 7% |
|  | Born at home | | 1% |
|  | Other ______________ | |  |
| ***8. In your opinion, dog is:***  ***(1= completely disagree and 5= completely agree)*** | A family member | 1 | 5% |
|  |  | 2 | 4% |
|  |  | 3 | 11% |
|  |  | 4 | 24% |
|  |  | 5 | 56% |
|  | Man’s best friend | 1 | 1% |
|  |  | 2 | 5% |
|  |  | 3 | 15% |
|  |  | 4 | 24% |
|  |  | 5 | 55% |
|  | Only an animal | 1 | 50% |
|  |  | 2 | 20% |
|  |  | 3 | 10% |
|  |  | 4 | 5% |
|  |  | 5 | 14% |
|  | A dangerous animal that spreads diseases | 1 | 67% |
|  |  | 2 | 17% |
|  |  | 3 | 9% |
|  |  | 4 | 6% |
|  |  | 5 | 1% |
|  |  | Other |  |
| ***Section 2*** | **COMMUNITY OWNED DOGS (CODs) AND HUMAN-ANIMAL RELATIONSHIP** | | |
| ***9. Do you know about the existence of CODs in the Abruzzo region and that figure is regulated by a regional law?*** | Yes | | 41% |
|  | No | | 59% |
| ***10. There are CODs in the residence area in which You live?*** | Yes | | 24% |
|  | No | | 55% |
|  | No-opinion | | 21% |
| ***11. If you answered question number 10 “yes”, what do you think about CODs?*** | They are too many of them: | Yes | 29% |
|  |  | No | 65% |
|  |  | No-opinion | 6% |
|  | They make public areas dirty (faeces/urine): | Yes | 33% |
|  |  | No | 45% |
|  |  | No-opinion | 22% |
|  | They are annoying (when barking) and dangerous for the people (bites, car accidents, etc.): | Yes | 19% |
|  |  | No | 66% |
|  |  | No-opinion | 15% |
|  | They make me feel safe: | Yes | 28% |
|  |  | No | 58% |
|  |  | No-opinion | 14% |
|  | They are an added value for the community: | Yes | 45% |
|  |  | No | 27% |
|  |  | No-opinion | 27% |
|  | It’s better a free dog than a dog in a shelter: | Yes | 63% |
|  |  | No | 21% |
|  |  | No-opinion | 16% |
|  | I think they could be better managed: | Yes | 58% |
|  |  | No | 16% |
|  |  | No-opinion | 26% |
|  | I feel compassion due to the fact that they live in the street: | Yes | 56% |
|  |  | No | 33% |
|  |  | No-opinion | 11% |
|  | Other _________________ | |  |
| ***12. Do you happen to deal with any CODs, even though they are not in your residencial area?*** | Yes | | 35% |
|  | No | | 64% |
| ***13. Just in case you have ticked “yes” in 12. In your opinion, the health status of the CODs with whom you have been in contact appears:*** | Very good | | 5% |
|  | Good | | 36% |
|  | Sufficiently well | | 37% |
|  | Poor | | 17% |
|  | No-opinion | | 5% |
| ***14. If you tick “yes” in question 12, do you feel threatened when CODs come close to you?*** | Yes | | 7% |
|  | Sometimes, it depends on the dog’s behaviour (if it growls or looks aggressive) | | 27% |
|  | Sometimes, it depends on the dog’s size | | 22% |
|  | Never | | 43% |
| ***15. If you tick “yes” in question 12, when CODs are approached by You, or other people, they express:*** | Happiness/playfulness | | 23% |
|  | Indifference | | 27% |
|  | Aggression | | 9% |
|  | Fear | | 2% |
|  | They would like to be stroked | | 38% |
|  | Other_____________________ | |  |
| ***16. Is there anyone who takes care of CODs***? (if there is any) | Yes | | 40% |
|  | No | | 9% |
|  | No-opinion | | 51% |
| ***17. If you tick “yes” in question 16, do you take care of your residencial area’s CODs yourself?*** | Weekly | | 21% |
|  | Monthly | | 29% |
|  | Never | | 50% |
| ***18. If you answered “never” in question 17 go straigh to question 21.***  ***If you take care of CODs, what kind of help do You offer?***  Multiple choice allowed | Food | | 42% |
|  | Shelter | | 12% |
|  | Veterinary care (and food) | | 3% |
|  | No assistance | | 18% |
|  | Other___________________________ | |  |
| ***19. Why do you take care of CODs?*** | I love animals | | 82% |
|  | Nobody takes care of them | |  |
|  | Authorities don’t deal with them | | 4% |
|  | I feel compassion for them | |  |
|  | I think it’s important to be directly involved in useful activities in my area | | 13% |
|  | Other _________________ | |  |
| ***20. How does it make you feel taking care of CODs?*** | It makes me feel good | | 67% |
|  | I feel appreciated also by other people | |  |
|  | I think it’s ethically right | | 10% |
|  | I don’t mind | | 23% |
|  | Other_________________________ | |  |
| ***Section 3*** | **CODs AND STRAY DOG POPULATION CONTROL** | | |
| ***21. What do you think about CODs as a measure to control the stray dog population?*** | I support it | | 54% |
|  | I am against it | | 29% |
|  | No-opinion | | 10% |
|  | Other ________________________________ | | 8% |
| ***22. In case you have answered “I am against it”: how do you think the stray dogs and the problem of dogs’ abandonment should be faced?*** | By birth control | | 42% |
|  | By promoting dogs’ registration | |  |
|  | By building of new shelters | | 6% |
|  | By promoting adoption and responsible ownership | | 48% |
|  | No-opinion | | 4% |
|  | Other __________________________ | |  |
| ***23. In your opinion, could CODs be a problem for your fellow-citizens?*** | Yes | | 38% |
|  | No | | 22% |
|  | No-opinion | | 40% |
| ***24. In your opinion, could CODs be a problem for*** | Personal safety | Yes | 21% |
|  |  | No | 67% |
|  |  | No-opinion | 21% |
|  | Public health | Yes | 19% |
|  |  | No | 73% |
|  |  | No-opinion | 7% |
|  | Environmental hygiene | Yes | 30% |
|  |  | No | 50% |
|  |  | No-opinion | 20% |
|  | Other animals’ health and safety | Yes | 24% |
|  |  | No | 54% |
|  |  | No-opinion | 21% |
| ***25. Have you ever seen or learnt about inappropriate behaviours (e.g. mistreatment, poisoning) towards CODs?*** | Yes | | 24% |
|  | No | | 76% |
| ***26. If you have ticked “yes” in question 25, do you know if legal actions against the responsible have been taken?*** | Yes | | 21% |
|  | No | | 49% |
|  | No-opinion | | 31% |
| ***27. Do you think that a COD would live better if adopted by a family/person?*** | Yes | | 83% |
|  | No | | 4% |
|  | No-opinion | | 13% |
| ***28. Do you believe people should be educated and involved in issues regarding CODs?*** | Yes | | 83% |
|  | No | | 2% |
|  | No-opinion | | 15% |

**Table S2.** Association between the respondents who consider CODs a problem for the personal safety (question 24), the Public health (question 24.1), the environmental hygiene (question n.24.2), the other animals’ health and safety (question n.24.3) and their residence area .

| **Respondents residence area** | **In your opinion, could CODs be a problem for the personal safety?** | | **In your opinion, could CODs be a problem for the Public health?** | | **In your opinion, could CODs be a problem for the environmental hygiene?** | | **In your opinion, could CODs be a problem for the other animals’ health and safety?** | |
| --- | --- | --- | --- | --- | --- | --- | --- | --- |
|  | No | Yes | No | Yes | No | Yes | No | Yes |
| Country side | 19 | 19 | 27 | 6 | 8 | 13 | 19 | 14 |
| Town/Village | 251 | 65 | 254 | 65 | 168 | 105 | 185 | 74 |
| Outskirt | 56 | 15 | 68 | 19 | 64 | 23 | 51 | 23 |
| Chi squared | 16,67 | | 0,21 | | 10,00 | | 2,68 | |
| *p*-value | **0,0002** | | 0,9014 | | 0,0067 | | 0,2614 | |

**Table S3.** Association between the respondents who consider CODs a problem for the personal safety (question 24), the Public health (question 24.1), the environmental hygiene (question n.24.2), the other animals’ health and safety (question n.24.3) and their pet ownership.

| **Do you own a pet?** | **In your opinion, could CODs be a problem for the personal safety?** | | **In your opinion, could CODs be a problem for the Public health?** | | **In your opinion, could CODs be a problem for the environmental hygiene?** | | **In your opinion, could CODs be a problem for the other animals’ health and safety?** | |
| --- | --- | --- | --- | --- | --- | --- | --- | --- |
|  | No | Yes | No | Yes | No | Yes | No | Yes |
| Dog | 198 | 58 | 214 | 37 | 141 | 68 | 145 | 58 |
| Dog and Cat | 3 | 3 | 5 | 1 | 1 | 5 | 1 | 5 |
| Cat | 23 | 7 | 27 | 15 | 24 | 18 | 21 | 16 |
| Other animals | 20 | 3 | 25 | 3 | 13 | 4 | 18 | 2 |
| None | 82 | 30 | 78 | 36 | 61 | 48 | 70 | 33 |
| Fisher exact test | | | | | | | | |
| *p*-value | 0,3386 | | **0,0004** | | **0,0234** | | **0,0234** | |
